# SIN-3 as a key determinant of lifespan and its sex dependent differential role on healthspan in C*aenorhabditis elegans*

**DOI:** 10.18632/aging.101682

**Published:** 2018-12-12

**Authors:** Renu Pandey, Meenakshi Sharma, Daman Saluja

**Affiliations:** 1 Dr. B. R. Ambedkar Centre for Biomedical Research, University of Delhi, Delhi -07, India; * Equal contribution

**Keywords:** *C. elegans*, healthspan, hermaphrodite, mitochondrial dysfunction, SIN-3

## Abstract

Aging/senescence includes not just decline in lifespan but also etiologies of age associated morbidities which are inadequately understood. Extensive research has been undertaken to delineate the pathways and generate mutants with extended lifespan. However, little is known about the health status of these long lived mutants in the background of important genetic perturbations. *Caenorhabditis elegans* is one of the leading *in vivo* model organisms to study aging. Deletion of SIN-3, a transcription coregulator in *C. elegans* has been shown to reduce the lifespan of the mutant worms by half as compared to the wild-type and isogenic controls. The current study focuses on the effect of SIN-3 deletion on the healthspan of the worms. We find that not only are *sin-3* mutants more susceptible to stress, but the overall stress intolerance and physiological decline is sex dependent. The severity of the phenotype is more pronounced in hermaphrodites as compared to the males carrying the same mutation with respect to the controls. The results further suggest that genetic perturbation along with the gender play an important role in determining the lifespan, healthspan and overall fitness of an organism.

## Introduction

Lifespan analysis as a measure of aging has been a conventional parameter explored by bio-gerontologists. Aging itself is not a disease but is associated with an increased burden of diseases [1]. Technological and biological advancements in the last two-three decades have been successful in enhancing the lifespan considerably. But mere enhancement in lifespan or longevity does not translate into a healthy aging as the quality of life declines and organism becomes more susceptible to late-life illness [2,3]. Therefore, a paradigm shift is warranted in terms of more focus on research towards betterment of the health conditions of the elderly primarily by adding *more life to years* rather than mere *more years to life* strategy and secondly, by evaluating the neglected gender specific/based predisposition to diseases and their etiology.

Aging studies done in experimental organisms have given insight into the pathways such as TOR, dietary restriction and Insulin/IGF signaling as well as genes like sirtuins and protein deacetylase that have conserved effect across the species from yeast to mammals [4–7]. Further studies done in long-lived mutants of reduced insulin/IGF signaling showed delayed onset of diseases in both worms and mouse model system [8]. *Caenorhabditis elegans* is one of the most extensively used model organism for studying aging. Behavioral parameters such as pharyngeal pumping, swimming, movement assays and reproductive phase etc. related to healthspan and muscle health are well characterized in the worm. Chronological correlation between the healthspan to lifespan extension in various long-lived mutants of *C. elegans* such as *daf-2, eat-2, ife-2 and clk-1* suggested that the mutations merely prolonged aging without any benefits in the healthspan [9]. However, the usage of any uniform metric for estimating healthspan in invertebrates is still controversial [10,11].

No study has taken the sex differentiation and its effect on health during aging. We therefore, were interested to analyze the healthspan of short lived *sin-3* mutant of *C. elegans* [12] and understand if the gender/sex plays a determining role in accelerating or influencing the predisposition to aging morbidities.

Swi-independent-3 (Sin3), initially identified in budding yeast as a large acidic protein (~174.9 kDa) [13,14], is a global regulator of transcription for several genes involved in diverse biological processes both as a negative and positive regulator [15–17]. Sin3 protein is characterized by multiple paired amphipathic α-helix (PAH), HDAC interacting domain (HID) and a highly conserved region (HCR). The variability of the proteins interacting with the core SIN3/ Histone deacetylase (HDAC) complex is responsible for the wide repertoire of activities shown by the core complex both as co-repressor and co-activator [12,18–21]. SIN3/SIN-3 is evolutionarily conserved across different phyla and has different isoforms, we performed various behavioral assays to ascertain the role of *sin-3* in developmental and physiological processes as well as to establish its sex influenced role in determination of healthspan in *C. elegans.* Another advantage of using *C. elegans* to study SIN-3 is the presence of its single isoform [22] which not only makes the mutation more pronounced but also prevents accumulation of background effects due to the cross-reactivity of the isoforms. The parameters to estimate the healthspan in *C. elegans* vary and there is no consensus for the same. Keeping this in view we have tried to score maximum behaviors in *C. elegans* to get the clear picture of healthspan in *sin-3* mutants. The present study gives us an insight into the SIN-3 mediated effect on longevity of nematodes and helps to establish SIN-3 as a major determinant of gender-specific healthspan. Based on this study we conclude that loss of *sin-*3 not only causes reduction in lifespan but also compromises the healthspan and the effect of *sin-3* deletion is more pronounced in a gender specific manner, and is essential for the overall healthy maintenance of nematodes.

## RESULTS

Elucidation of longevity fails to determine quality of life and at times has been misinterpreted to imply that mutations cause increase or decrease in lifespan without paying attention to preserving the quality of life. The assays to determine healthspan in an invertebrate model system are still controversial and range from their physiological response to biochemical assays. Thus, we decided to evaluate the healthspan of *sin-3* mutation and elucidate the effect on health status of the worms using multiple parameters.

### Diminished muscle function and decreased lifespan is an outcome of *sin-3* deletion in *C. elegans*

**Figure 1:**
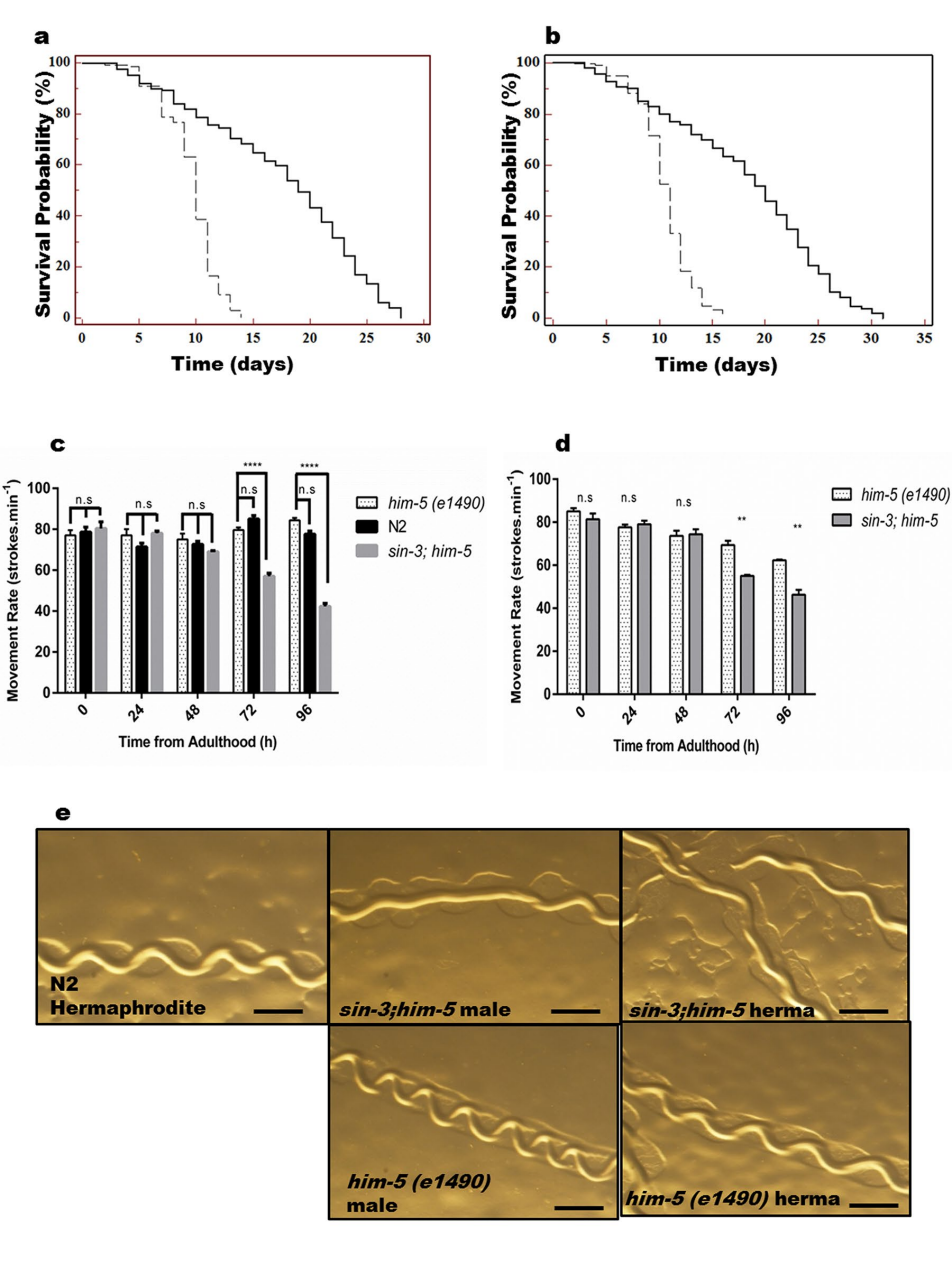
***sin-3* deletion causes reduction in lifespan.** Kaplan-Meier survival curve showing the lifespan of *sin-3;him-5* (dotted lines) and *him-5(e1490)* (solid lines) worms in (**a**) hermaphrodites and (**b**) males. Both hermaphrodites and males with *sin-3* deletion manifest significant reduction in mean lifespan. (**c**) Movement rate in *sin-3;him-5* hermaphrodites is compromised. Body bends are represented as strokes per minute. 20 hermaphrodites per strain were evaluated and the experiment was repeated thrice. (**d**) Movement rate in *sin-3;him-5* males is compromised as compared to *him-5* mutant males. Body bends are represented as strokes per minute. 20 males per strain were evaluated and the experiment was repeated thrice. (**e**) Pictures representing the tracks formed by nematodes of strains mentioned. (ns, non-significant; **p < 0.05; ****p < 0.001 and denotes the comparison with respect to *him-5 (e1490)*; One-way ANOVA performed). The images were captured using Nikon stereoscopic zoom microscope at 5X zoom. Scale is equivalent to 200 pixels.

Earlier results from our lab demonstrated that wild-type worms fed with bacteria expressing dsRNA against s*in-3* gene show significantly reduced lifespan. We further decided to study the role of *sin-3* deletion mutant worms [*sin-3(tm1276);him-5(e1490)* hereafter referred as *sin-3;him-5*] in comparison to the isogenic control worms [*him-5(e1490)* hereafter referred as *him-5*] on lifespan and muscle function. The lifespan of *sin-3;him-5* hermaphrodite worms was significantly reduced with mean and maximum lifespan of 10 and 14 days respectively as compared to 19 and 28 days in *him-5* hermaphrodite worms (Figure 1a). In males mean and maximum lifespan observed in *sin-3;him-5* was 11 and 16 days respectively as compared to 20 and 30 days in *him-5* worms (Figure 1b and Tables S1 and S2).

Like in humans, aging in *C. elegans* coincides with muscle deterioration, or sarcopenia, which is associated with reduced muscle function [23]. Muscles in *C. elegans* lack regenerative potential in the adult. Therefore, it offers a unique opportunity to study how different stress insults affect aging in a non-regenerating tissue. Several studies have demonstrated age associated decline in coordinated motility and function during *C. elegans* aging [24–26]. Since movement is an index of vitality and muscle frailty (a known marker for aging) we decided to look for any locomotory defects arising in the background of *sin-3* mutation. In preliminary experiments, we found that nematodes are capable of normal sinusoidal movement till day 10 of adulthood. As *C. elegans* ages, a dramatic decrease in swimming ability, pharyngeal pumping and maximum movement velocity has been previously reported [10,27] and decline in these physiological behaviours is the hallmark of overall muscle health and indicate muscle frailty with progression in age of *C*. *elegans*. Our data demonstrated that movement rate (measured in strokes/minute) of *C. elegans* harbouring *sin-3* deletion was significantly compromised with age in hermaphrodite (57 ± 1.5 strokes/ min) as compared to its isogenic (79 ± 1.2 strokes/minute) and wild-type (85 ± 1.7 strokes/minute) controls at 72 h and is reduced by approximately 50% at 96 h (Figure 1c). Similar results were obtained when wild-type worms were fed dsRNA against *sin-3* (Table S2).

Parallel trend was observed in males where the mean strokes per minute were 55 ± 0.6 and 69 ± 2.2 respectively for *sin-3;him-5* deletion mutant and *him-5* mutant at 72 h; and 46 ± 2.1 and 62 ± 0.3 respectively at 96 h (*p* < 0.05). Interesting observation was that males of isogenic control worms with genotype *him-5* manifested significant reduction in number of body bends/minute as compared to their hermaphrodite counterpart both at 72 and 96 h (Figure 1d and S1a).

Another important assay to determine the muscle health is pharyngeal pumping and surprisingly we did not observe any significant difference in both *sin-3;him-5* and *him-5* mutants with age (Figure S1b). When tracks formed by the mutant worms w.r.t controls were observed at young adult stage, we found that *him-5* and wild-type N2 worms gave perfect sinusoidal track with average crest and trough of 0.19 ± 0.03 mm for *him-5* and 0.26 ± 0.02 mm for N2 hermaphrodite. Similar trend was observed for males of *him-5* where average crest and trough was measured to be 0.18 ± 0.04 mm. However, due to presence of uncoordinated tracks in both *sin-3;him-5* hermaphrodite and males, determination of crest and trough was not possible and the tracks were almost straight (Figure 1e and S1c). Our observations further revealed that *him-5* worms were fast moving whereas *sin-3;him-5* worms were slow moving with a velocity of 0.25 ± 0.02 mm s^-1^ as compared to 0.58 ± 0.05 mm s^-1^ in *him-5* worms (Figure S1d).

### *sin-3* deletion causes remarkable differences in physiological parameters in *C. elegans*

**Figure 2:**
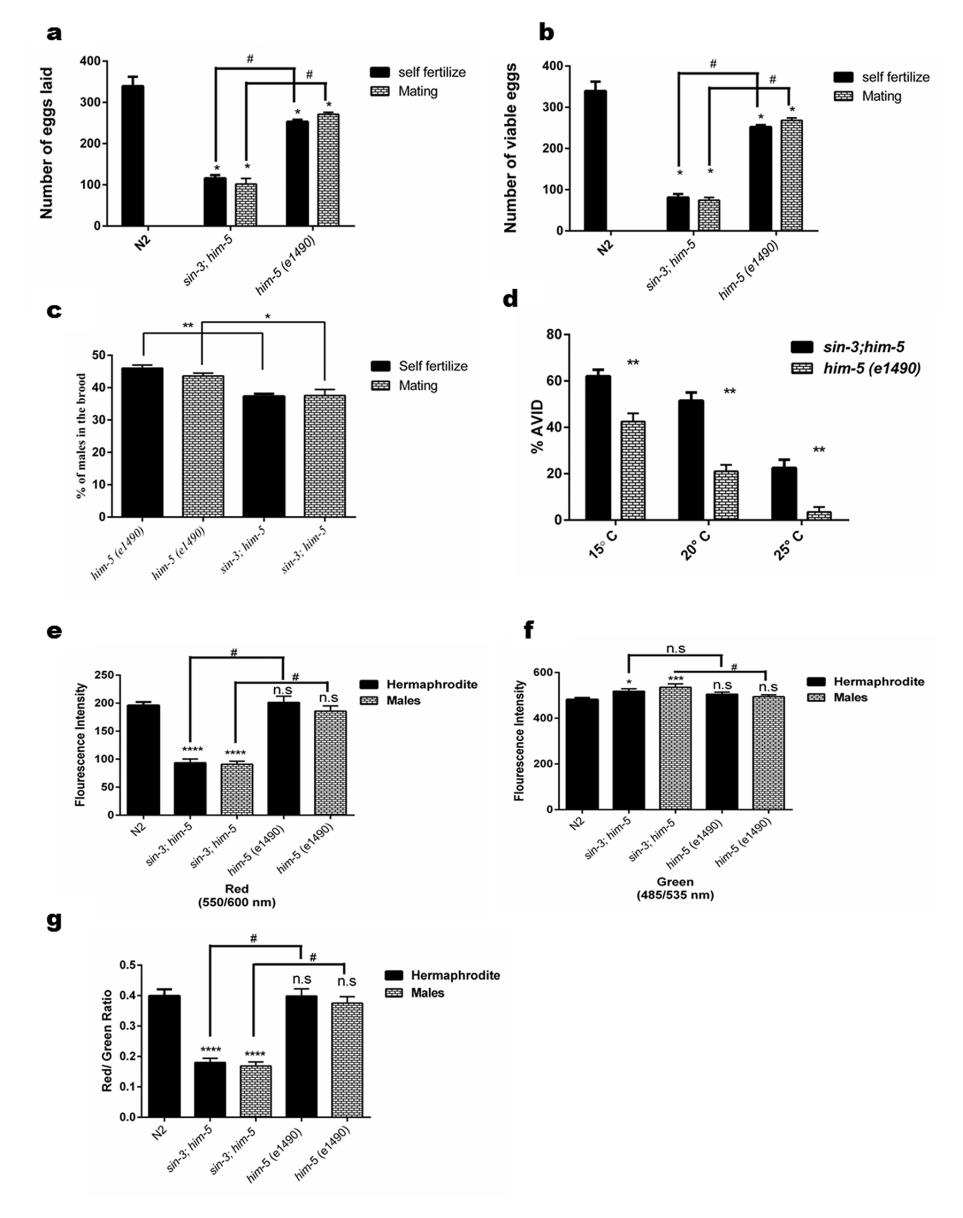
**Loss of *sin-3* causes reproductive defects in *C. elegans.*** Graphical representation of (**a**) total number of eggs laid in the entire reproductive span of worms, (**b**) total number of eggs that were viable and (**c**) percentage of males in the viable progeny after self-fertilization and mating. (**d**) Loss of *sin-3* causes AVID phenotype defect in *C. elegans* measured as percentage of worms showing ruptured vulva and protrusion of intestine and uterus. (**e-g**) Graphical representation for the quantification of red, green and the ratio of red/green for JC-1 staining respectively depicting the hypopolarisation of mitochondrial membrane potential in case in *sin-3* deletion in *C. elegans* (For figure a, b and e-f: ns, non-significant; *p < 0.05; **p < 0.001, ****p < 0.0001 and denotes the comparison with respect to wild-type N2; Two way ANOVA performed # *p <*0.001 denotes the comparison between *sin-3;him-5* and *him-5(e1490)* Student’s t test performed. For figure (c) and (d) *p < 0.05; **p < 0.001, ****p < 0.0001 two way ANOVA performed).

The reproductive ability of the organism may also be a reflection of both the lifespan and healthspan. When we assessed the reproductive ability of *sin-3;him-5* worms, we found that the *sin-3;him-5* worms demonstrated reduced brood size. The total number of eggs produced by *sin-3;him-5* worms in self-progeny were 115.8 +3.48 and the viable eggs were 81.2 + 3.7 as compared to 253.2 + 2.1 total eggs and almost all (252.2 + 2.1) eggs were viable in *him-5* worms (Figure 2a and 2b). The number of males in progenies were almost similar whether the hermaphrodites were allowed to mate or self-fertilize in both *sin-3;him-5* worms and isogenic control *him-5* worms (Figure 2c).

When the crossed progenies were observed, we found that *sin-3;him-5* worms produced 101.8 + 6.1 and the *him-5* worms produced 271.2 + 2.1 eggs respectively (Figure S2). The percent hatching of self-progeny and mated worms was observed to be 70% & 73% in *sin-3;him-5* and 99% in *him-5* worms (Table S3). Similar results were obtained when *him-5* worms were fed dsRNAi (Table S2).

Age associated vulval integrity defect (AVID) has been chosen as powerful marker for worm healthspan [28]. It can be defined as loss of vulval integrity; thereby ejection of internal organs occurs causing pre-mature death in *C. elegans*. These worms are often censored from lifespan experiments as being ruptured worms. Our results clearly show that the *sin-3;him-5* worms have increased AVID phenotype at 20 °C when compared to *him-5* worms. We observed about 18-20% worms showing AVID phenotype in *him-5* worms whereas *sin-3;him-5* worms showed 48-50% AVID phenotype at 20 °C (*p* < 0.05). The percentage of AVID increased with decreasing temperature and were 68-70% in *sin-3;him-5* worms as compared to 43-46% in *him-5* worms at 15 °C (Figure 2d). Similar observations were made with dsRNAi fed *him-5* hermaphrodites (Table S2).

### Mitochondrial functioning is compromised in *sin-3* deletion mutants

We have previously reported that intracellular oxidative stress is the underlying cause for enhanced autophagy and reduced lifespan [12]. In this study so far, we have observed that the *sin-3* mutant worms demonstrate an age associated decline in cuticle integrity, stress tolerance, altered physiological parameters, protein homeostasis, and muscle integrity in addition to reduced lifespan. It is reported that sustained high levels of intracellular ROS cause extensive macromolecular damage which induce p53 dependent apoptosis and affect the lifespan and cause accelerated aging and decline in healthspan [29]. To check the role of ROS if any, in *sin-3* mutants, mitochondrial membrane potential was determined using JC-1 staining of worms at young adult and at day 10 of attaining adulthood. The results clearly demonstrated that *sin-3;him-5* worms had higher membrane depolarization as evident by the perturbed Red::Green fluorescence intensity as compared to the wild-type N2 and the isogenic control. The mitochondrial defects were presented both by the hermaphrodite as well as the male of the *sin-3;him-3* strain (Figure 2g and Figure S3). This was further substantiated by the observation that *sin-3;him-5* worms showed significantly reduced total cellular ATP levels in both hermaphrodite as well as male populations at day 10 of adulthood with respect to the isogenic strain (Figure S4).

### *sin-3* deletion results in cuticle defects in *C. elegans*

**Figure 3:**
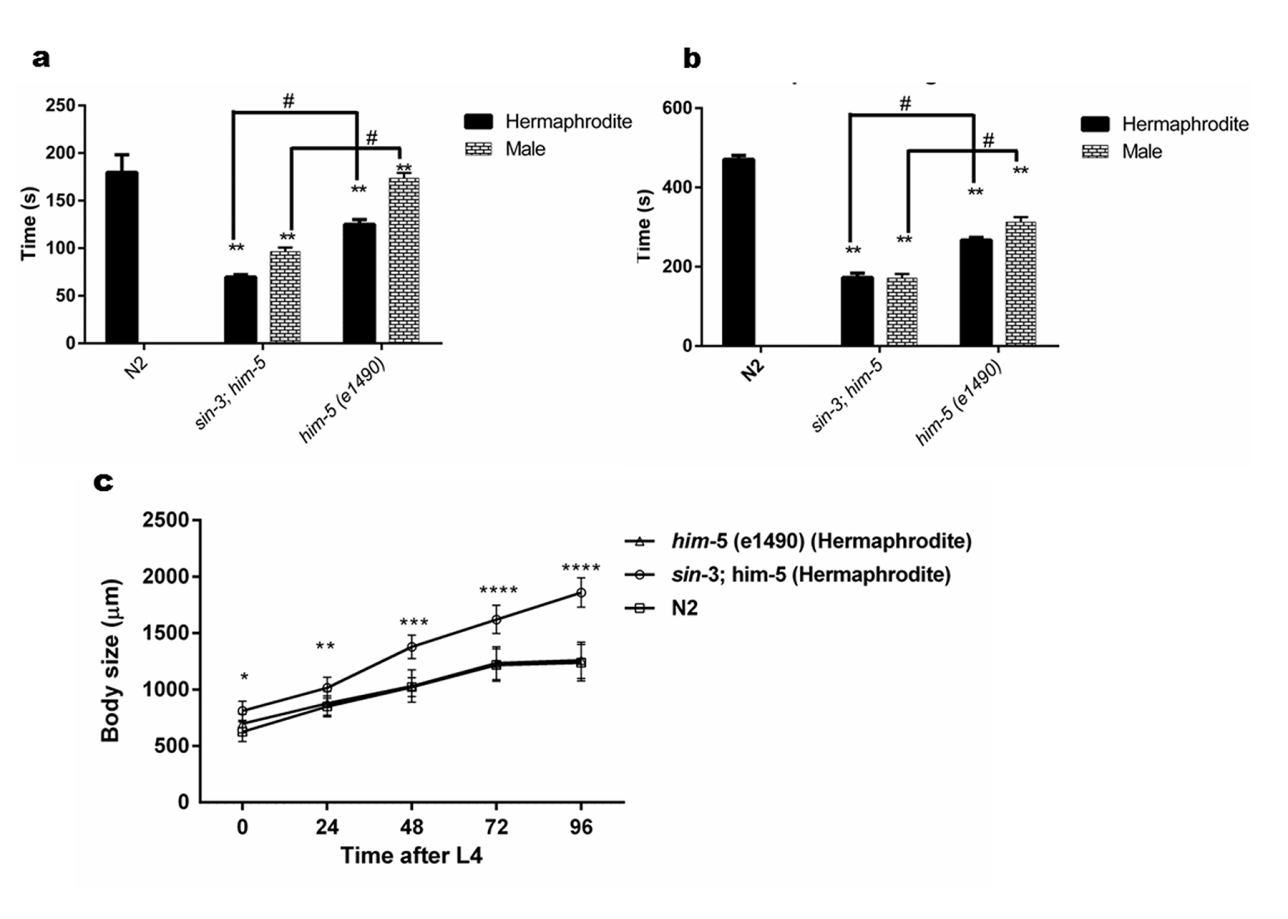
***sin-3* mutants have defective cuticle.** (**a**) Graph representing the time in seconds when first major break in the cuticle is observed. (**b**) Graph representing time in seconds till complete disintegration of the cuticle occurred. (**c**) Body length of *sin-3* mutant worms is longer. Graph representing the body length of nematodes of various strains measured at indicated time after attaining L4 stage (two way ANOVA was performed for all experiments; # *p < 0.001* denotes comparison w.r.t *him-5(e1490)* and ** *p < 0.001* represent comparison w.r.t N2).

**Figure 4:**
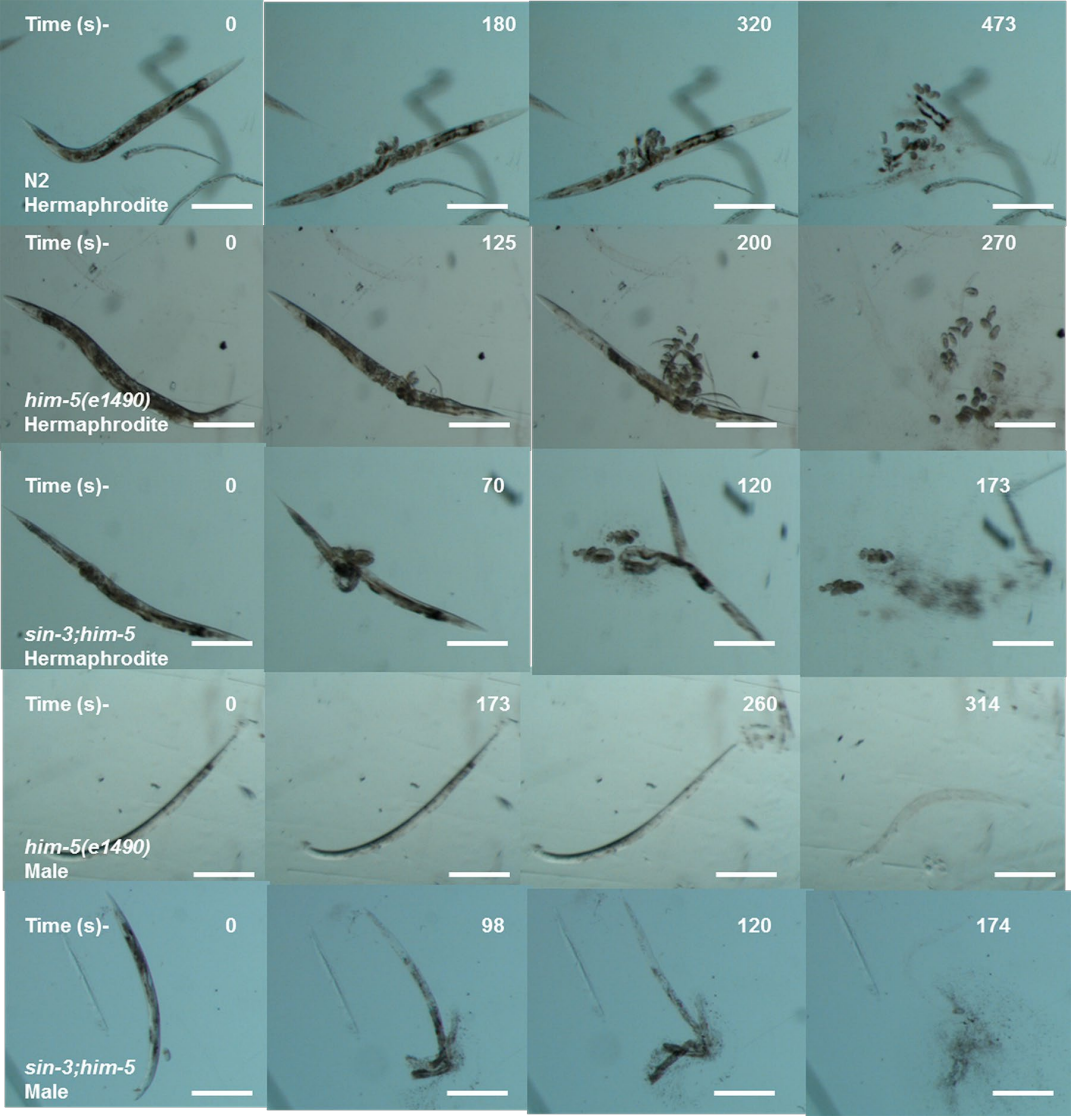
**Cuticle disintegration is rapid in *sin-3;him-5* mutants.** The representative images depicting time in seconds for first major break in cuticles (second row) till complete disintegration of the cuticle (last row) for *sin-3;him-5* hermaphrodites and males as compared to isogenic control *him-5(e1490)* and wild-type worms.

*C. elegans* have an outer body wall made up of collagen, called the cuticle that encapsulates the entire organism. This cuticle provides protection to the worm from outer environment and is also necessary for movement [30]. To analyze the health of the worm in relation to cuticle integrity, young adult worms grown on NGM plate were observed for first break in the cuticle when suspended in alkaline hypochloride solution. Our results show that *sin-3* mutants have defective cuticle when compared to their isogenic controls. The major break in cuticle was observed at 69.6 ± 1.45 s in *sin-3;him-5* hermaphrodite worms as compared to 125 ± 2.89 s in *him-5* worms. Similarly in males of *sin-3;him-5* worm the major cuticle break was at 96.7 ± 2.4 s as compared to 173.77 ± 3.2 s in *him-5* worms (Figure 3a and Figure 4). The total disintegration of cuticle was observed at the mean time of 172.6 ± 11.0 s for *sin-3;him-5* hermaphrodite worms and 267 ± 6.42 s in *him-5* worms; for males the time was 171.33 ± 10.26 s in *sin-3;him-5* worms and 312.67 ± 12.70 s in *him-5* worms (Figure 3b and Figure 4).

Since body size shows most noticeable change in the hermaphrodite we further decided to measure the body size as a function of time after young adult stage. Surprisingly the body length of *sin-3;him-5* worm was 32% longer than *him-5* and N2 worms after 96 h following young adult (YA) stage. Difference in body length was apparent from young adult stage worms where in the body length of *him-5* was 15% longer as compared to that of *sin-3;him-5* worm (Table S4). Similar observations were recorded in dsRNAi (against *sin-3)* fed *him-5* hermaphrodites (Table S2). The percentage body length increased with time in the *sin-3;him-5* hermaphrodites as by 48 h it had increased by 25% from 15% seen in L4 staged worms (Figure 3c and S5). The wild-type and *him-5* hermaphrodites too demonstrated an increase in body size with time. The increase in body size stagnated at 72 h after L4 stage in all the worms (Table S4).

### Stress tolerance and protein homeostasis is compromised in *sin-3* deletion mutants of *C. elegans*

**Figure 5:**
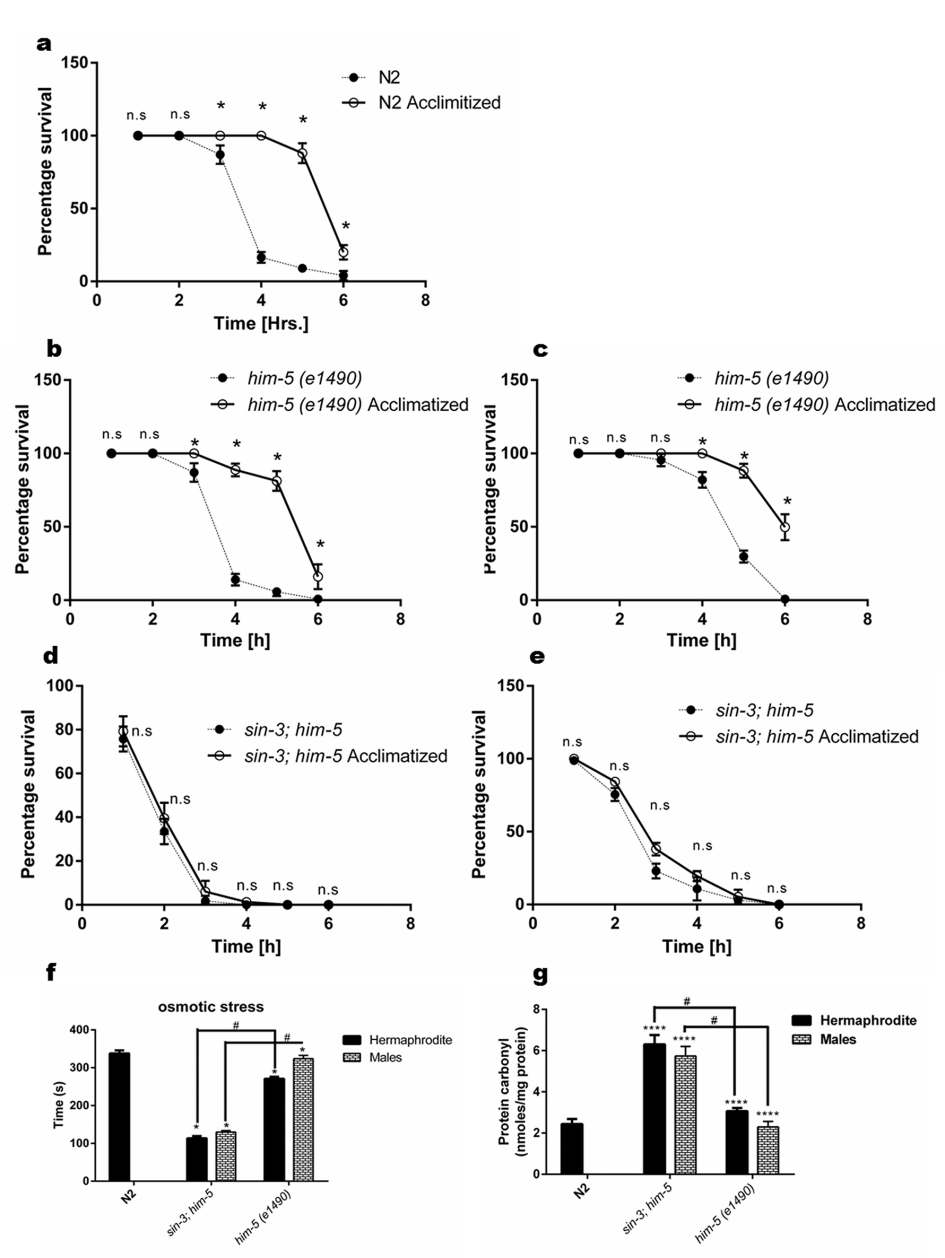
**Loss of *sin-3* causes decline in percentage survival of worms after thermal stress insult.** Percentage survival with and without acclimatization at 25 °C of (**a**) N2 hermaphrodite (**b**) *him-5(e1490)* hermaphrodites and (**c**) *him-5(e1490)* males. (**d**) Loss of *sin-3* causes decline in percentage survival after thermal stress insult in both hermaphrodites and (**e)** males (ns, non-significant; * *p <* 0.05; Student’s t test was performed). (**f**) Loss of *sin-3* causes decline in the survival after osmotic stress insults in the strains indicated when tested on NGM plates with 500mM NaCl. (**g)** *sin-3* deletion causes significant protein carbonylation. Hermaphrodite and males of the indicated strains were subjected to quantification of protein carbonyl content (ns, non-significant; *p < 0.05; **p < 0.001, ****p < 0.0001 and denotes the comparison with respect to wild-type N2; Two way ANOVA performed # *p <*0.001 denotes the comparison between *sin-3;him-5* and *him-5(e1490)* Student’s t test performed).

Clegg and coworkers [31] suggested that there is loss of ability to recover from internal and external stress in elderly people. *C. elegans* can be used to check the ability to maintain homeostasis in healthy animals which is measured as an organism’s ability to respond to various physiological perturbations [9]. We tested the tolerance of worms to two types of stress i.e. heat and osmotic stress in age synchronized young adult worms. The results demonstrated the isogenic control *him-5* hermaphrodite worms showed 50% mortality after 5 h of being subjected to heat stress after acclimatization and 4 h when exposed directly to heat stress (Figure 5b). Wild-type N2 hermaphrodites showed similar trend as that of the *him-5* hermaphrodites and showed 50% mortality by 3 h when directly subjected to heat stress while 5 h if acclimatization was done before heat stress (Figure 5a). In contrast, the *sin-3;him-5* hermaphrodite worm irrespective of acclimatization (25 °C for 18 h) followed by exposure to 37 °C, showed 50% mortality within 1 h of being exposed to the heat stress (Figure 5d).

We also observed that the *sin-3;him-5* males were more resistant to heat stress than hermaphrodites and were able to tolerate the heat stress for 3 h under both conditions (Figure 5e). In any case *sin-3;him-5* males were more sensitive compared to their isogenic *him-5* control males as latter survived up to 6 h when acclimatized and 4 h when exposed directly to heat stress (Figure 5c).

Another stress to which we exposed the worms to check their ability in maintaining homeostasis was osmotic stress; to that end we exposed the worms to 500 mM NaCl. The *sin-3;him-5* worms both hermaphrodite and males were significantly more sensitive to osmotic stress as compared to *him-5* and N2 worms. *sin-3;him-5* hermaphrodites survived the osmotic stress for 113.4 ± 3.6 s and males survived 129.8 ± 4.3 s as compared to the hermaphrodites and males of *him-5* worms surviving 270.8 ± 8.11 s and 323.8 ± 5.24 s respectively (Figure 5f).

Severity, specificity, site of generation and the kind of ROS imposed, determines the primary cellular target of oxidative stress dependent damage. Various kinds of protein modification can be brought about either directly or indirectly through reaction of secondary by products of ROS with amino acids. Methionine and cysteine residues are more prone to attack by almost all ROS species [32]. Indeed *sin-3;him-5* hermaphrodite and males show enhanced protein carbonylation (6.3 ± 0.23 nmoles/mg protein and 5.73 ± 0.27 nmoles/mg protein respectively) as compared to *him-5* hermaphrodite (3.07 ± 0.07 nmoles/mg protein) and males (2.3 ± 1.25 nmoles/mg protein) and wild-type N2 worms (2.43 ± 1.43 nmoles/mg protein) at YA stage (Figure 5g).

### Age associated pigments are accumulated in the *sin-3* deletion mutants

**Figure 6:**
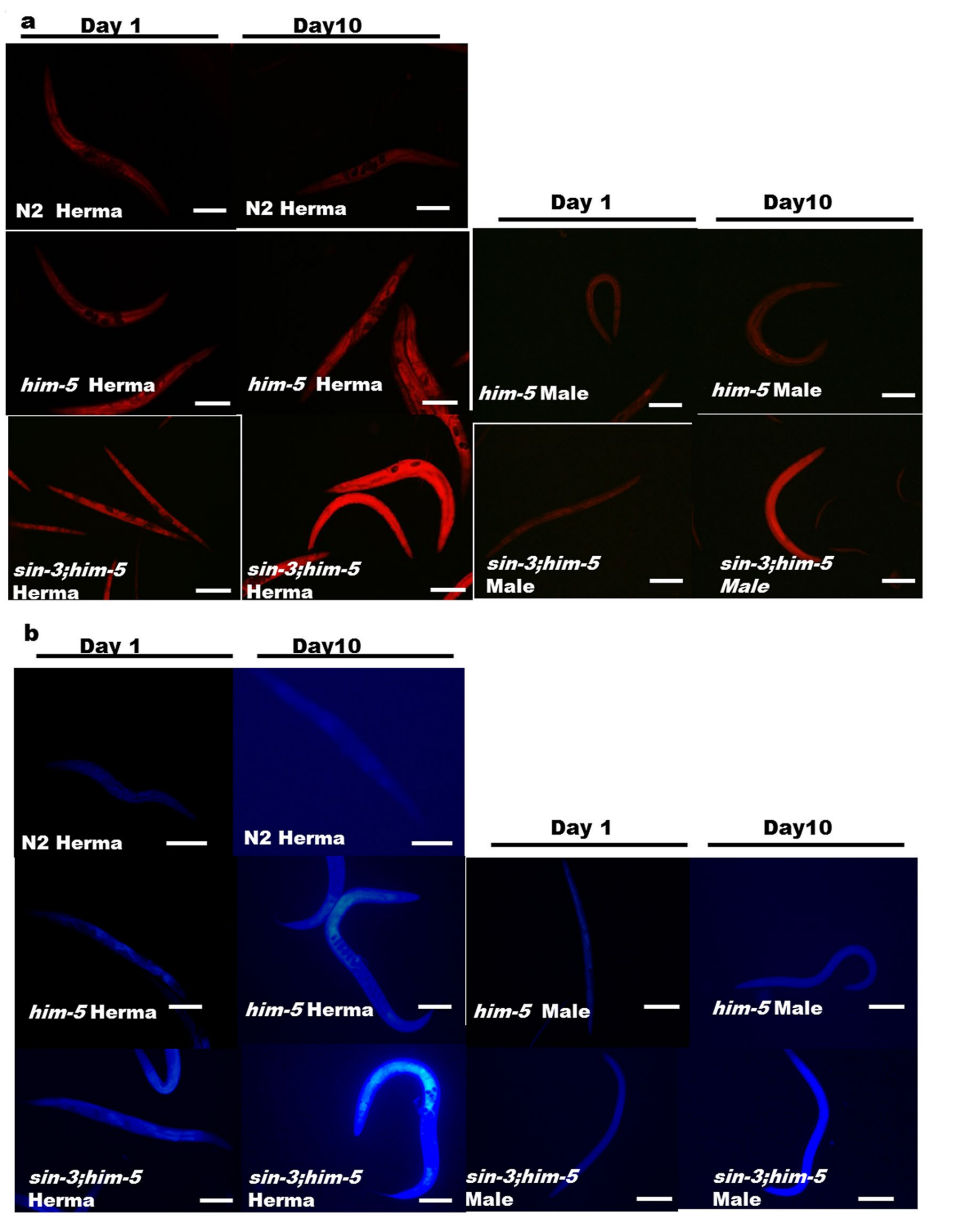
**Enhanced age associated lipid and lipofuscin deposition in the *sin-3;him-5*mutants** (**a**) *sin-3;him-5* hermaphrodite and male as compared to worms from *him-5(e1490)* and wild-type N2 worms at day one and day ten of attaining adulthood. (**b**) Enhanced age associated death pigment lipofuscin was observed in the *sin-3;him-5* hermaphrodite and male as compared to worms from *him-5(e1490)* and wild-type N2 worms at day one and day ten of attaining adulthood. Visualization was done using NIKON fluorescence microscope. Magnification 400X, scale: 500 µm. 30 worms per strain were visualized and the experiment was repeated thrice.

Our previous study strongly suggests that intracellular oxidative stress is the underlying cause for enhanced autophagy and reduced lifespan in *sin-3* mutants [12]. The enhanced autophagy motivated us to check other age related pigments which may be altered in *sin-3;him-5* worms. Nile staining of hermaphrodites and male worms demonstrated both qualitative and quantitative increase in age associated lipid deposition in *sin-3;him-5* mutants as compared to that of *him-5* and wild-type N2 strain with progressive aging from day1 to day 10 of adulthood (Figure 6a and S6). We also recorded the age associated death pigment lipofuscin and observed increased pigment in *sin-3;him-5* hermaphrodite and male as compared to the *him-5* isogenic control worms and wild-type N2 strain (Figure 6b and S6) with age.

## DISCUSSION

Aging in an organism is represented by a composite array of physiological deterioration which invariably does not correlate with extension in lifespan. Thus, the notion of ‘healthspan’, the functional and disease-free period spanning an organism's lifespan, certainly is an important determinant of the aging process [33]. Therefore, we assessed the impact of gender on *sin-3* mediated reduction in lifespan on the overall health status of the *sin-3;him-5* mutant worms. Aging is linked to time dependent decline in both motor function and structure of muscles. Previous reports have demonstrated Sin3A and Sin3B dependent regulation of a cohort of genes involved in sarcomere assembly and muscle contraction in mice attributing muscle defects to ablation of Sin3 [34]. Our results too demonstrate *sin-3* mediated progressive decline in muscle integrity with age. Not only do the *sin-3;him-5* mutants exhibit uncoordinated movement as demonstrated by unsynchronized tracks but also show advanced deterioration in the thrashing frequency (swimming ability) as early as 72 h of gaining adulthood in both the sexes as compared to the isogenic *him-5* worms. This implies that effect of *sin-3* deletion on muscle integrity is equally debilitating in both the sexes. Our results are in concordance with studies in *Drosophila* where *Sin3A* knockdown flies were lethargic and showed defects in locomotor activity [35].

Though sarcomere integrity is compromised in *sin-3* mutants, no significant difference in the pharyngeal pumping rate of *sin-3* mutants is observed in either hermaphrodite or males. One possible explanation could be that pharynx of the *C. elegans* shares similarity with mammalian heart as both are comprised of electrically coupled muscle cells [36,37]. Pharynx of the *C. elegans* has a well-defined nervous system; a network of 14 different types of neurons make up the neuromuscular feeding apparatus independent of the extra-pharyngeal nervous system. All chemical synapses onto pharyngeal muscle are formed by these neurons [38]. Since pharynx is under neuronal control, it shows no evident defect in the rhythmic pumping in the absence of *sin-3.* This is additionally supported by our observations that both *sin-3* mutants and their isogenic controls avoid high osmotic region suggesting that the sensory neuronal network is not affected in *sin-3* deletion worms (Figure S7). Thus, though muscle frailty increases with age in *sin-3* mutant worms, pharyngeal pumping rates are maintained.

*sin-3* mutant worms exhibit reproductive defects. The brood size is low along with decline in the percentage viability of the eggs laid. These results are in synchrony with earlier reports where deletion of either *prdx-2* or the cytosolic catalase *ctl-2* in worms, causes manifestation of progeric phenotype [39,40]. Our previous results also show defective catalase expression and activity in *sin-3* mutants indicating chronic peroxide stress [12]. Deletion of *sin-3* causes shortening of lifespan in *C. elegans,* providing evidence that *sin-3* mediated ROS induced damage contributes towards aging and lifespan. However, these results are in glaring contrast to studies where deletion of *sod-1* or *sod-*2 in *C. elegans* demonstrated extension in lifespan in spite of having high oxidative damage as evaluated by protein carbonylation content [41,42]. These results thrust open a possibility that hydrogen peroxide, superoxide and their subsequent reaction by products play distinct and vital role in aging and lifespan determination. The AVID phenotype data clearly suggests that the vulval integrity is significantly compromised in *sin-*3 mutant hermaphrodites. Since vulva is lined with cuticle secreted by hypodermis [43], reproduction and ensuing damage to the vulva may affect the outcome of the stress experiments. To prevent the experimental bias arising out of compromised vulva in stress tolerance and cuticle integrity, the stress related experiments were performed using age synchronized young adult worms.

*sin-3* mutant worms present severely compromised cuticle integrity and stress tolerance as compared to isogenic controls. Previously Barnes and coworkers [35] had reported that male and female *Drosophila* flies with ubiquitous knockdown of *Sin3A* showed increased sensitivity to paraquat induced oxidative stress. Adding to these observations, our results show *sin-3* mutant hermaphrodites and males exhibit significant decline in heat and osmotic stress tolerance. Acclimatization of the *sin-3* mutants at 25 °C did not show any beneficial effect towards tolerance of subsequent heat stress which was evident in the control worms. This can be explained as genetic screens have implicated Sin3-Rpd3 complex as an essential axis to the heat stress response in yeast through the Msn2/4 transcription factors [44]. Though *C. elegans* does not harbor Msn2 or Msn4 homolog, PANTHER database denotes *lin-26* as the ortholog of Msn4 [45]. LIN-26 encodes a zinc-finger protein with a unique C2H2 motif and along with this, other C2H2 proteins, LIR-1, LIR-2, and LIR-3 are essential during *C. elegans* development [46]. Therefore, thermal stress intolerance observed in *sin-3* mutants could be due to yet unidentified *lin-26* route similar to the behavior observed in yeast cells. The *sin-3* dependent decline in osmotic stress tolerance may be attributed to mitogen activated protein kinase (MAPK), Hog1, which recruits Sin3/Rpd3 complex on the promoters of osmo-stress responsive genes in a manner similar to Msn2/4 to regulate survival of yeast under osmotic stress stimuli [47]. Further experiments are warranted to ascertain the molecular pathways leading to heat and osmotic stress intolerance in *C. elegans*.

In agreement with the results obtained in *Drosophila* cell line where Sin3 deletion leads to aberrant mitochondrial function, perturbed ATP levels and upregulation of nuclear and mitochondrial genes [48], we observed *sin-3;him-5* worms had significantly reduced total cellular ATP levels in both hermaphrodite as well as male populations with respect to the isogenic control. The mitochondrial membrane potential too is perturbed as JC-1 staining confirmed hypopolarisation of the mitochondrial membrane potential indicating a loss in mitochondrial integrity and predisposition to DNA damage and apoptosis. We have previously reported that autophagy is significantly enhanced in worms fed with bacteria expressing dsRNA against *sin-3* gene [12]. Sin3/Rpd3 complex is essential not only for mitochondrial sustenance but also regulates mitophagy by repressing the expression of Atg32 in yeast cells which in turn assures longevity and cellular lipid homeostasis [49]. Importantly, *sin-3* mutants show enhanced lipid droplet accumulation which is not observed in isogenic control, suggesting defective lipophagy. Protein carbonyl content along with intracellular accumulation of auto-fluorescent compound, lipofuscin in *sin-3;him-5* at a significantly higher rate than the controls indicate heightened cellular oxidative damage, deteriorating health status and accelerated aging in *sin-3* mutants.

In conclusion, we summarize that most of the physiological and behavioral defects observed in *sin-3* mutants are in concordance with the previously implicated cellular processes that are peroxide sensitive. Complementing the high intracellular oxidative stress in the background of *sin-3* deletion [12], the worms also demonstrate a sex influenced decline in stress tolerance, protein homeostasis, respiration, mitochondrial function and muscle integrity. Deletion of *sin-3* further leads to accumulation of lipids indicating a defective lipophagy and enhanced age associated death pigments like lipofuscin. We suggest that the pleotropic effect of the oxidative damage precipitates the dysfunction in cellular homeostasis leading to reduction in both health status and lifespan of the worms. This study helps in unraveling the pleotropic effect of SIN-3 corepressor in overall maintenance of the well-being of the *C. elegans* and its role in regulation of lifespan and sex influenced effect on the healthspan of the worms. Our results support that SIN-3 is the pivot in this redox-longevity-healthspan axis and further experiments are warranted to obtain better insights in unraveling how sex influences the effects at molecular level.

## MATERIALS AND METHODS

### *C. elegans* strains and culture conditions

The strains N2 Bristol (wild-type), *sin-3* deletion mutant strain KC565 (*sin-3(tm1276)*I*; him-5(e1490)*V), CB4088 *him-5(e1490)V* and *E.coli* OP50 were procured from the Caenorhabditis Genetics Center (CGC, University of Minnesota). All strains were maintained as described by Brenner [50]. Briefly, nematode growth media (NGM) was prepared and worms were fed with *E. coli* strain OP50 at 20 °C unless otherwise stated. For all experiments young adult synchronous population, obtained by bleaching as described previously [12] were used unless otherwise stated. When performing dsRNAi experiments, young adults (P0) were fed with bacteria expressing the desired RNAi and the L3 progeny of the F2 generation were examined. For lifespan analysis and measurement of Age associated vulval integrity defects (AVID) worms were picked and allowed to lay eggs to avoid stress conditions.

### Age synchronization

Age synchronization was performed as standardized previously [12]. Briefly, egg-bearing worms fed on OP50 were collected using M9 buffer from NGM plates. Gravid adult worms were bleached/lysed in the bleaching solution, followed by a quick centrifugation at 1300 g. The pellet consisting of worm debris was then washed with M9 buffer at least three times. The eggs so obtained were then resuspended in M9 buffer and the tubes were incubated overnight at 20 °C with fairly vigorous shaking to obtain synchronous L1 worms.

### Analysis of lifespan

Adults were hand-picked and transferred to fresh plate seeded with the OP50 bacteria for egg laying. The adult worm was removed after eggs were laid and the plates were incubated to obtain worms at L4 stage. The worms were segregated into hermaphrodite and male worms for the lifespan analysis at 20 °C. In all the experiments, the pre-fertile period of adulthood (L4 stage) was used as t = 0 for lifespan analysis. The animals that ruptured, bagged (exhibited internal progeny hatching), or crawled off the plates were censored from lifespan analysis data. Each lifespan experiment was repeated at least three times with n = 75 to 100 worms per experimental group for both males and hermaphrodites. Kaplan-Meier survival analysis was used to compare the mean lifespan of different treatments and *P*-values were calculated using the log rank (Mantel-Cox method).

### Measurement of AVID

Avid measurements were carried out as previously described [28]. Briefly, gravid adults were placed on NGM plates seeded with *E. coli* OP50 lawns for egg-laying. Subsequently, adults were removed from the plates, and eggs were allowed to hatch and reach L4 stage. Male worms were removed and the experiment was continued with hermaphrodites. Worms were transferred daily till their reproductive phase and later periodically to prevent starvation and avoid contamination. The AVID state was noted for each hermaphrodite under a stereo zoom microscope (Nikon SMZ1000). Worms that crawled off the plate were not considered.

### Disintegration assay

Cuticle disintegration was measured as previously described by Watts and coworkers [51]. Briefly, age synchronized young adult nematodes were placed in 0.5 ml alkaline hypochlorite solution (1% sodium hypochlorite, 0.25 M NaOH) in a 96-well transparent bottom plates and cuticle disintegration was scored the time taken for the first major break in the cuticle. Plates were agitated gently every 20 s during observation. Error bars are plotted as mean ± SEM. For each experiment, fifteen worms were taken and the experiments were repeated at least thrice.

### Stress assay

***Osmotic stress.*** Resistance to osmotic stress was assessed as described earlier [52], by transferring age synchronized young adult worms to NGM plates containing 500mM NaCl. Twenty animals per plate were scored for survival every 2 minutes till they showed no movement due to dehydration induced paralysis. Results are plotted as mean ± SEM and statistical significance was calculated using two way ANOVA (*P≤ 0.05* was considered significant; Bonferroni test was used for multiple comparison).

***Thermo tolerance assay.*** Thermo tolerance assays were conducted at the indicated temperature (37 °C) and live worms were scored every hour for 6 h. For acclimatization, age synchronized young adult worms were first kept at 25 °C for 18 h before shifting to 37 °C. Plates were initiated with 20-25 adult animals each and were placed in an incubator. All plates were numbered randomly and at least three biological replicates were used and then counted for survival every hour.

For all stress resistance assays, Day 1 young adult worms were used. We used at least three biological replicates with a minimum of 20 worms per replicate.

### Estimation of Brood size

Brood size was determined as described by Swain et al. [53]. Briefly, single L4 hermaphrodite animal alone or hermaphrodite with 4 males (for determining the brood size after mating) were placed onto individual plates. The plates were incubated at 20 °C and the worms (P0) were transferred to a new plate (replica-plated) every 24 h until they stopped laying eggs, and the number of eggs was recorded every day. Viable progeny was determined by counting the number of hatched larvae (F1) the day following transfer. The nematode brood size was determined based on the sum of total eggs laid by individual hermaphrodites. Percentage of males in the brood was determined after 72 hours by identifying and counting the number of males in F1 progeny using a Nikon SMZ1000 stereo zoom microscope.

### Body length, body width and tracks of young adult worms

To measure the body length, L4 larvae worms with an obvious white crescent surrounding the visible prospective vulva were chosen and picked onto fresh plates and incubated at 20°C for 24 h to develop into young adults. 20 worms were photographed per strain using Nikon SMZ1000 stereoscopic zoom microscope. All length measurements were performed with the free Java image processing program ImageJ [54]. Young adult worms were measured from the nose to the tail tip. The body widths were measured at the position of the vulva, from side to side.

To photograph worm tracks, single young adult worms were moved to new plates containing a one day old bacterial lawn and were left undisturbed for 10 minutes. 20 worms per strain per assay were used and the experiment was repeated thrice.

### Movement/Thrashing assay

Body bending thrashing assay was performed as described previously [55]. Briefly, 35 mm diameter sterile NGM agar plates were filled with 1 ml of M9 buffer. Before the start of assay, worms were put on a sterile NGM agar plate without bacteria and allowed to crawl freely to remove the agglomerated bacteria from the worm. After examining visually whether the bacteria were removed, young adult (day-1 adult) worm were put into the buffer and allowed to swim freely for 1 minute to be accustomed to the environment. The observations were similarly recorded for each worm after the indicated time from adulthood. Number of body thrashes was counted for one minute. 20 worms per strain per assay were counted. A movement of the worm that swings its head and/or tail to the same side is counted as one thrash.

### Pharyngeal pumping assay

Pharyngeal pumping was recorded everyday along with lifespan from adult day one until death of the worms [56]. The pumping assays were performed on NGM agar plates at room temperature using Nikon SMZ1000 stereo zoom microscope. Pharyngeal pumping was defined as the number of times the terminal bulb of the pharynx contracted over one-minute interval. All pumping rates were measured on a lawn of OP50 bacteria at 20 °C at indicated duration after attaining adulthood. Worms were placed on NGM/OP50 plates and left undisturbed for 1 hour before measuring. 20 worms per strain per assay were observed. The experiment was repeated at least thrice.

### ATP assay

The ATP assay was performed as described before, with minor modifications [57]. Age synchronized young adult worms were grown on NGM plates seeded with OP50 and collected for assay. In all, 200 age-synchronized day 10 adult worms of various strains were washed with M9 buffer, treated with four freeze-thaw cycles by dipping in liquid N2. The worms were then homogenized in ATP assay buffer followed by deproteinization using 4M Perchloric acid (Fisher Chemicals, A2296, 70.5% w/w or ~11.7 M) and 2M KOH (Merck Millipore, CAS: 1310-58-3) method as described in the kit. Samples were then spun at 4 °C, 11,000 g for 10 minutes. A colorimetric/fluorometric ATP assay kit (Abcam, Cambridge, UK, AB83355), which utilizes the phosphorylation of glycerol to generate a product that is easily quantified by fluorometric (Ex/Em = 535/587 nm) methods, was used to quantify ATP contents. ATP concentrations were determined using standard curve derived from fluorescence of known ATP concentrations as per the kit instructions. Microplate fluorescence reader (Tecan infinite M200) was used to measure levels of fluorescence. For normalization, protein levels were determined by BCA protein assay kit (Pierce, Thermo Scientific, Rockford, IL, USA).

### Osmotic Avoidance assay:

Age synchronized day 10 adult worms were evaluated for osmotic avoidance behavior (OSM) as described previously [52]. The behavior was quantified as the number of worms that crossed a 2 cm ring of 8M glycerol in 10 minutes. 30 worms per strain were observed and the experiment was repeated thrice.

### Nile Red assay

The feeding Nile red assay was conducted by seeding wild-type or mutant age synchronized L1 *C. elegans* on NGM plates containing *E. coli* OP50 supplemented with 50 ng/mL Nile red (diluted fresh into 100 mL M9 media per plate from 500 mg/mL stock in acetone and added to the top of *E. coli* plates and allowed to dry). Worms were synchronized at 20 °C in minimal media to obtain the L1 staged worms. Imaging and quantification was conducted after growth at 20 °C as day one and day ten adults using a Nikon Eclipse E600 microscope fluorescence microscope at 200X. All Nile red analyses were carried out on animals grown at 20 °C. 30 worms per strain were visualized and the experiment was repeated thrice.

### Protein carbonylation

Worm homogenate was prepared as described by Sharma et.al., [12] and protein carbonylation was quantified using alkaline DNPH method as previously described [58]. Briefly, for each reaction, 400 µl of DNPH (10 mM in 0.5 M H_3_PO_4_) (2,4-Dinitrophenylhydrazine, Sigma, D199303) was mixed with 400 µl of protein solution containing 500 µg of protein (obtained by sonication of age synchronized day one young adult worms in PBS). The mixture (800 µl) was incubated in dark at room temperature for 10 minutes followed by incubation with 200 µl of NaOH (6 M) for 10 minutes. The absorbance was read at 450 nm with the UV spectrophotometer (Perkin Elmer, MA, USA). Blank contained 400 µl of 0.5M H_3_PO_4_ instead of DNPH. Molar absorption coefficient of 21 mM^−1^ cm^−1^was used and the carbonyl content was calculated as nanomoles of DNPH incorporated (protein carbonyls) per mg of protein.

### JC-1 staining for mitochondrial potential (ΔΨm)

The assay was performed as previously described [59]. Briefly, assay of ΔΨm was performed using JC-1 (5,5′,6,6′-tetrachloro-1,1′,3,3′-tetra-ethyl benzimid-azolylcarbocyanine iodide, catalog no. T-3168, Molecular Probes) dissolved in DMSO at a concentration of one mg/ml. Age synchronized day one and day ten hermaphrodite and males were suspended separately in PBS containing freshly prepared 2% paraformaldehyde and incubated for 20 minutes for fixation, followed by washing with M9 buffer. Samples were subsequently transferred to micro centrifuge tubes containing 1µM JC-1 reagent and incubated at 37 °C in a 5% CO_2_ incubator for 45 minutes. Samples were washed and suspended in S basal buffer, placed in 96 well black bottom fluorescence plates. Around 100 worms were used per well per replicate. Measurements of red fluorescence (excitation 550 nm, emission 600 nm) and green fluorescence (excitation 485 nm, emission 535 nm) were performed using a fluorescence plate reader (Tecan, infinite M200). The experiment was repeated thrice.

### Age pigment (Lipofuscin) Accumulation

One and ten days old adult worms were transferred onto freshly prepared 2% (w/v) agarose pads for microscopy. Levimasole (1mM) was added to the agarose pads to anesthetize the worms. Images were captured on a Nikon Eclipse E600 fluorescence microscope at 200X magnification. Lipofuscin levels were quantified after the acquired images were converted to 16-bit images, thresholded and subsequently quantified using ImageJ software by determining the average pixel intensity in each worm body. 30 worms per strain were visualized and the experiment was repeated thrice.

### Statistical Analysis

All data is expressed as mean ± SEM. Unless otherwise stated all data was analyzed using One-way ANOVA for multiple comparisons. Kaplan-Meier survival analysis was used to compare the mean lifespan of different strains. All histograms were generated and all statistical analysis were performed using GraphPad Prism (GraphPad Software Inc. version 6.01). Bonferroni's multiple comparisons test was employed and *P*< 0.05 was accepted as statistically significant difference. The results have been expressed as mean ± SEM unless otherwise stated.

## SUPPLEMENTARY MATERIALS

Figure S1

Figure S2

Figure S3

Figure S4

Figure S5

Figure S6

Figure S7

Supplementary Tables S1-S4
