# Supplementary material for: SIN-3 as a key determinant of lifespan and its sex dependent differential role on healthspan in Caenorhabditis elegans
Source: Aging (Albany NY). 2018 Dec 12;10(12):3910–37. doi: 10.18632/aging.101682 (PMC6326684; doi:10.18632/aging.101682)
Supplement: Figure S1 [file aging-10-101682-s001.pdf]

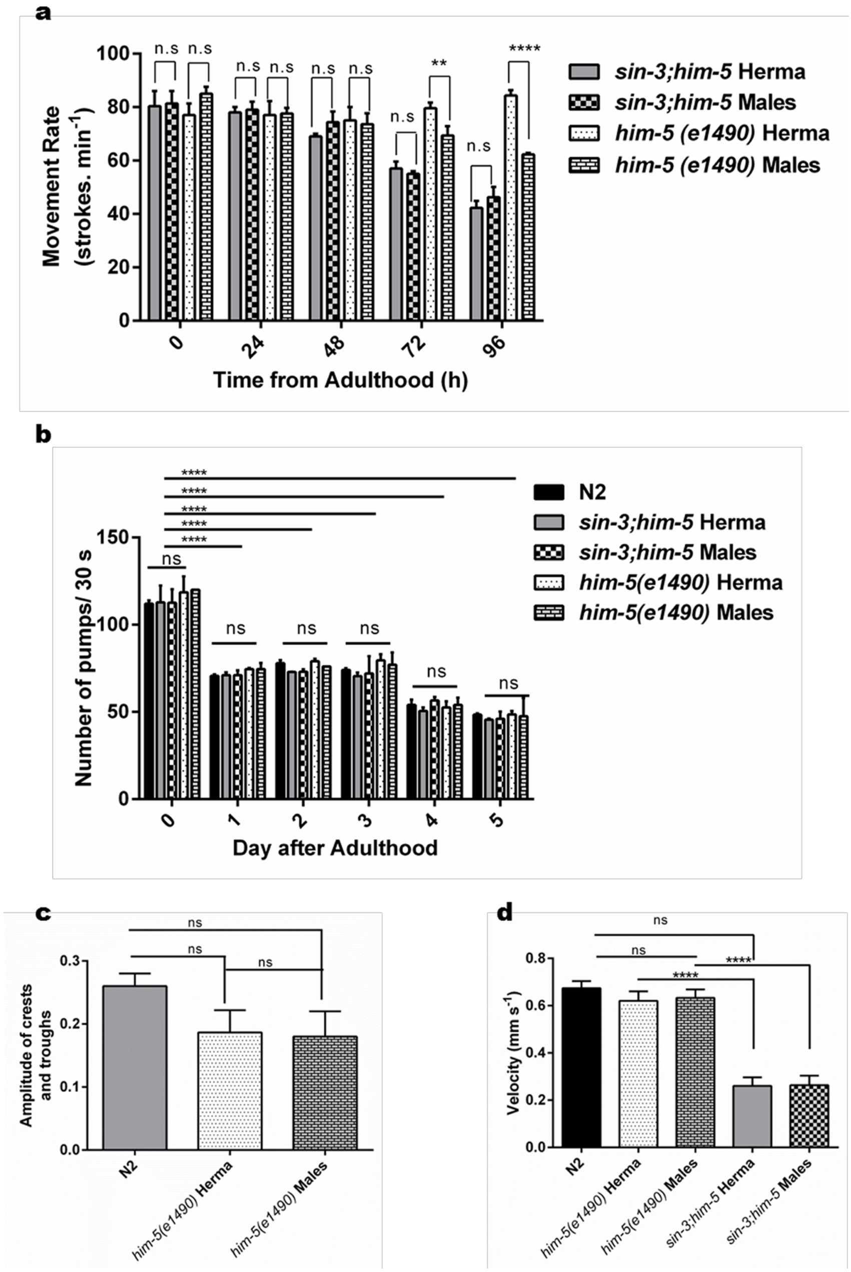

Figure S1. Diminished muscle function is an outcome of *sin-3* deletion. (**a**) Movement rate in *sin-3;him-5* hermaphrodite and males is not significantly varied however the decline in movement rate in *him-7(e1490)* males is more pronounced as compared to *him-5(e1490)* hermaphrodites. Body bends are represented as strokes per minute. (**b**) Effect of *sin-3* deletion on pharyngeal pumping. The decline in pharyngeal activity with age depicted as pumping or contradictions /30 s is observed in *sin-3;him-*5 hermaphrodites and ales as compared to the *him-5(e1490)* and wildtype worms. The pharyngeal contractions over time remains unaffected in all the worms compared. (**c**) The quantification of the tracks measured as crests and troughs formed by *sin-3;him-5* hermaphrodites and males as compared to isogenic control *him-5* mutant and wild-type worms. (**d**) the velocity measured in mm/ s for *sin-3;him-5* hermaphrodites and males as compared to isogenic control *him-5* mutant and wild-type worms (ns, non-significant; **$p < 0.05$; ****$p < 0.001$ and denotes the comparison with respect to *him-5 (e1490)*; One-way ANOVA performed). At least 20 worms per strain were evaluated and the experiment was repeated thrice.
