# Supplementary material for: SIN-3 as a key determinant of lifespan and its sex dependent differential role on healthspan in Caenorhabditis elegans
Source: Aging (Albany NY). 2018 Dec 12;10(12):3910–37. doi: 10.18632/aging.101682 (PMC6326684; doi:10.18632/aging.101682)
Supplement: Figure S2 [file aging-10-101682-s002.pdf]

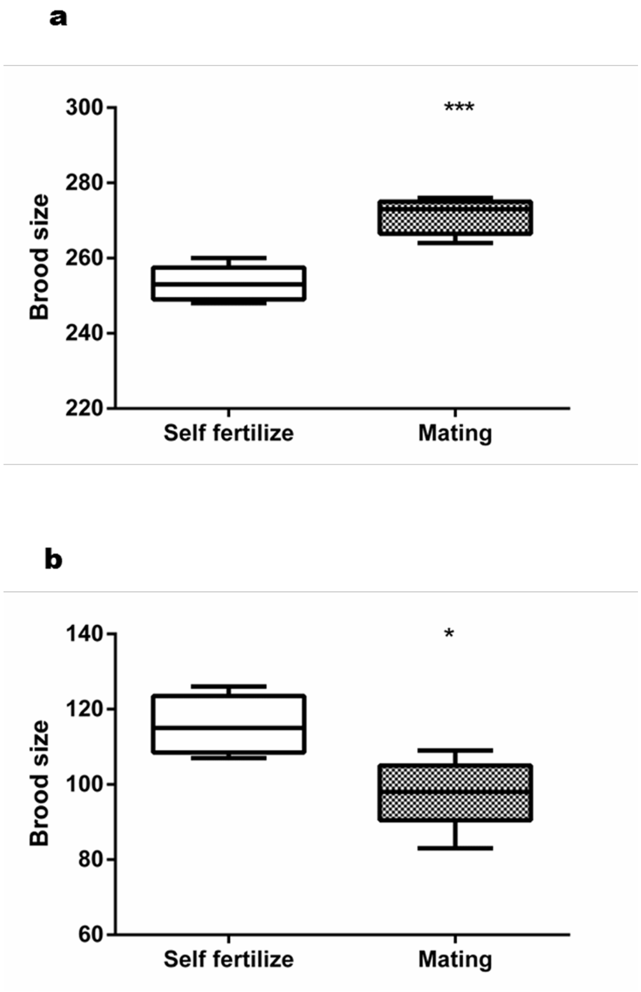

Figure S2. *sin-3* deletion causes reduction in the brood size. (**a**) *him-5(e1490)* hermaphrodite lays significantly greater number of eggs after mating as compared to self-fertilization. (**b**) *sim-3;him-5* hermaphrodite lays significantly reduced number of eggs after mating as compared to self-fertilization. (***P* < 0.05; ****P* < 0.001 and denotes the comparison with respect to self-fertilized brood size; paired Student's t-test performed). At least 30 hermaphrodites per strain were evaluated and the experiment was repeated thrice.
