# Supplementary material for: SIN-3 as a key determinant of lifespan and its sex dependent differential role on healthspan in Caenorhabditis elegans
Source: Aging (Albany NY). 2018 Dec 12;10(12):3910–37. doi: 10.18632/aging.101682 (PMC6326684; doi:10.18632/aging.101682)
Supplement: Figure S3 [file aging-10-101682-s003.pdf]

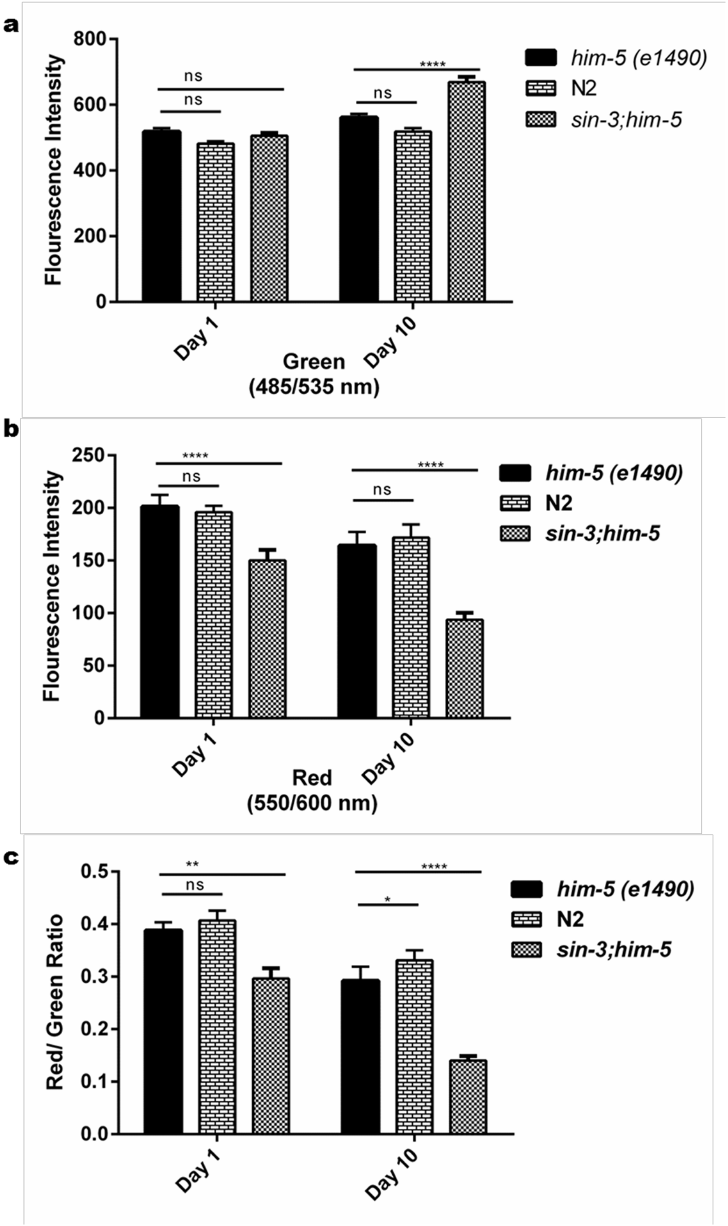

Figure S3. *sin-3* deletion causes decline in mitochondrial health. (**a**) *sin-3;him-5* worms show enhanced green fluorescence at day 10 as compared to *him-5(e1490)*and N2. (**b**) *sin-3;him-5* worms have lower red fluorescence at day 1 and day 10 as compared to *him-5(e1490)*and N2. (**c**) With age the ratio of red/green fluorescence is highly perturbed in *sin-3;him-5* worms as compared to the isogenic and wild-type worms (ns = non-significant; *$P < 0.05$; **$P < 0.01$; ****$P < 0.001$ and denotes the comparison with respect to self-fertilized brood size; two way ANOVA performed). The experiment was repeated thrice.
