# Supplementary material for: SIN-3 as a key determinant of lifespan and its sex dependent differential role on healthspan in Caenorhabditis elegans
Source: Aging (Albany NY). 2018 Dec 12;10(12):3910–37. doi: 10.18632/aging.101682 (PMC6326684; doi:10.18632/aging.101682)
Supplement: Figure S4 [file aging-10-101682-s004.pdf]

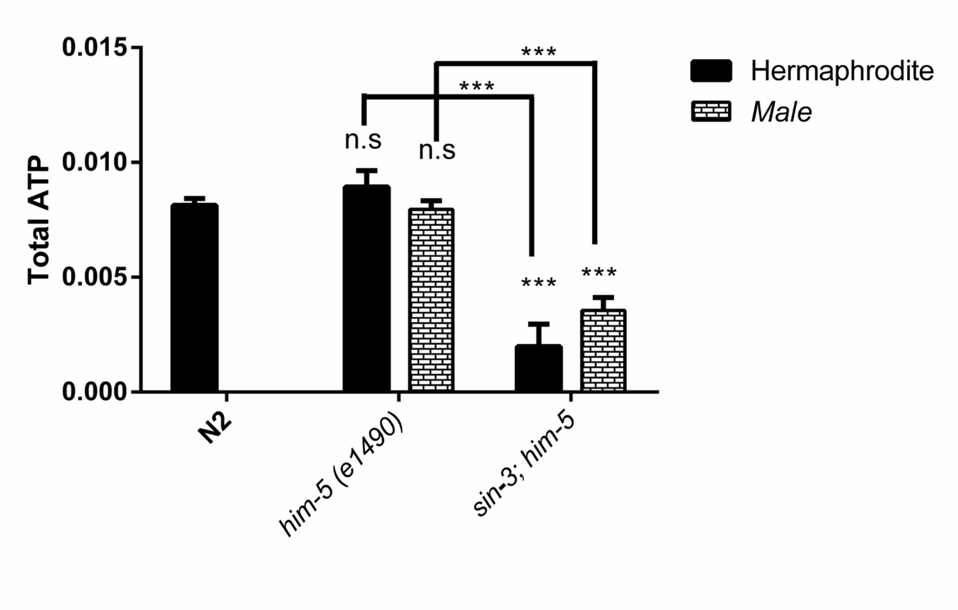

Figure S4. Total ATP content in *sin-3(tm1279);him-5(e1490)* worms is significantly reduced in both hermaphrodite as well as male populations with respect to the isogenic strain at day 10. (ns, non-significant; ***P* < 0.05; *****P* < 0.001 and denotes the comparison with respect to *him-5 (e1490)*; One-way ANOVA performed).
