# Supplementary material for: SIN-3 as a key determinant of lifespan and its sex dependent differential role on healthspan in Caenorhabditis elegans
Source: Aging (Albany NY). 2018 Dec 12;10(12):3910–37. doi: 10.18632/aging.101682 (PMC6326684; doi:10.18632/aging.101682)
Supplement: Figure S5 [file aging-10-101682-s005.pdf]

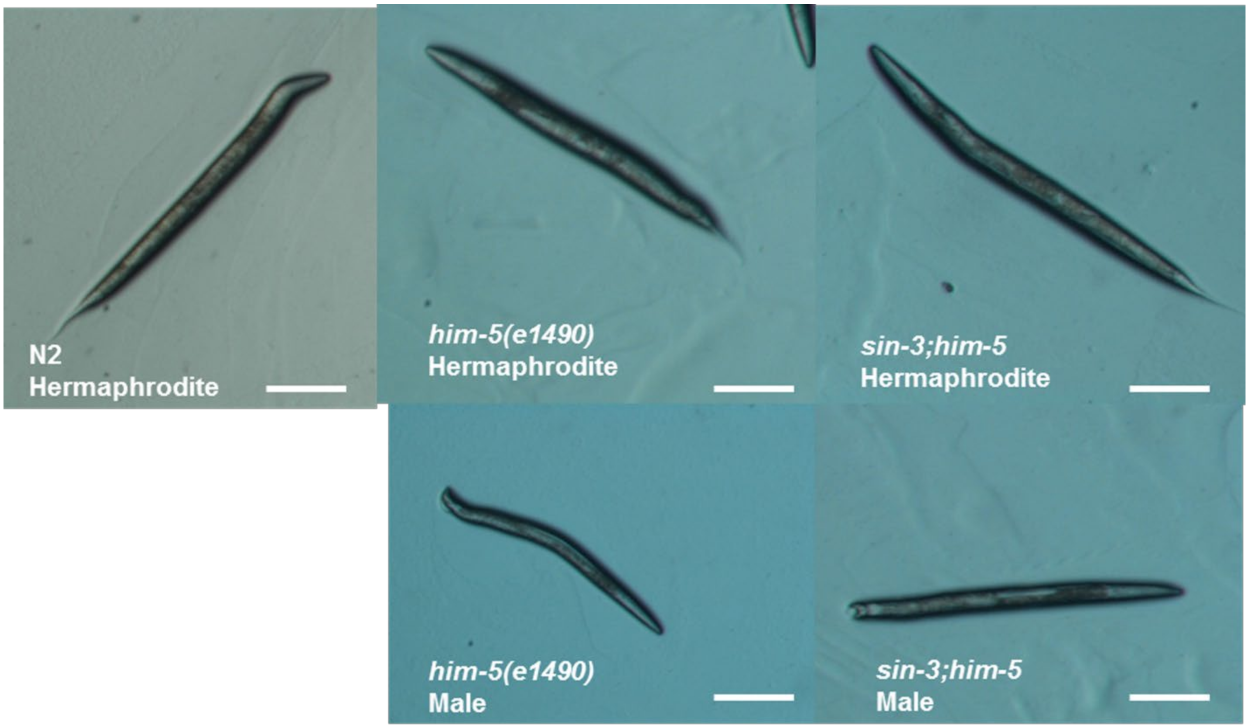

Figure S5. *sin-3;him-5* worms are longer than the isogenic and wild-type controls. The representative images of worms depicting body length of *sin-3* mutant worms as compared to the isogenic as well as wild-type worms at L4 stage. At least 20 worms per strain were evaluated and the experiment was repeated thrice.
