# Supplementary material for: SIN-3 as a key determinant of lifespan and its sex dependent differential role on healthspan in Caenorhabditis elegans
Source: Aging (Albany NY). 2018 Dec 12;10(12):3910–37. doi: 10.18632/aging.101682 (PMC6326684; doi:10.18632/aging.101682)
Supplement: Figure S6 [file aging-10-101682-s006.pdf]

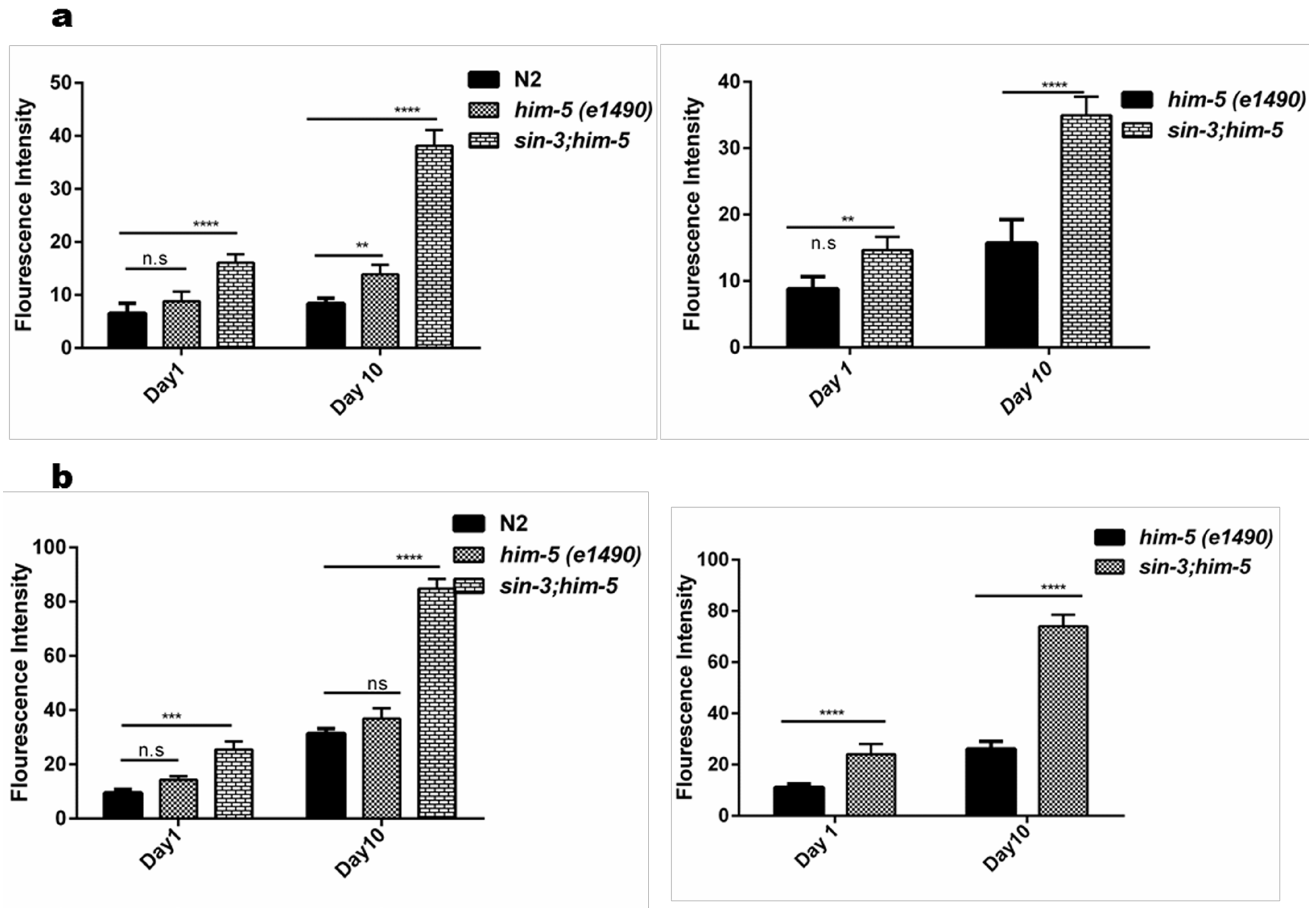

Figure S6. *sin-3* deletion causes accumulation of lipids and age associated death pigment lipofuschin. (**a**) *sin-3;him-5* worms show enhanced accumulation of lipids at day 10 of adulthood w.r.t isogenic control and wild-type. Left and right panel denotes the quantitative accumulation in hermaphrodites and males respectively. (**b**) *sin-3;him-5* worms show enhanced accumulation of lipofuschin at day 10 of adulthood w.r.t isogenic control and wild-type. Left and right panel denotes the quantitative accumulation in hermaphrodites and males respectively (ns = non-significant; *P < 0.05; **P< 0.01; ****P < 0.001 and denotes the comparison with respect to self-fertilized brood size; Two way ANOVA performed). The experiment was repeated thrice.
