# Supplementary material for: SIN-3 as a key determinant of lifespan and its sex dependent differential role on healthspan in Caenorhabditis elegans
Source: Aging (Albany NY). 2018 Dec 12;10(12):3910–37. doi: 10.18632/aging.101682 (PMC6326684; doi:10.18632/aging.101682)
Supplement: Figure S7 [file aging-10-101682-s007.pdf]

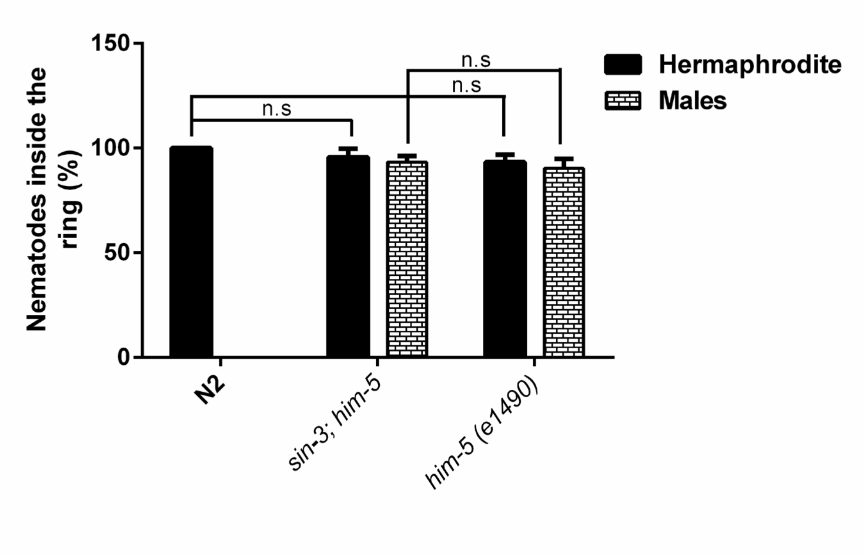

Figure S7. *C. elegans* assessed for aversion behavior using 8M glycerol in ring assay. The ability avoid high osmotic region was observed for all the strains. No significant difference observed. Each data point represents means ± the standard error of three replicates. (n.s denotes non-significant. Statistical significance was calculated comparing single treatments.)
