# Supplementary material for: SIN-3 as a key determinant of lifespan and its sex dependent differential role on healthspan in Caenorhabditis elegans
Source: Aging (Albany NY). 2018 Dec 12;10(12):3910–37. doi: 10.18632/aging.101682 (PMC6326684; doi:10.18632/aging.101682)
Supplement: Supplementary Tables S1-S4 [file aging-10-101682-s008.pdf]

Supplementary Tables

Supplementary Table S1. Decline in lifespan in *sin-3(tm1279);him-5(e1490)* worms compared to *him-5(e1490)* mutant worms.

Genotype	Mean lifespan	% decline in mean lifespan	Maximum lifespan	% decline in maximum lifespan
**Hermaphrodite**				
him-5(e1490)V	19	N.A	28	N.A
*sin-3(tm1276)*I; *him-5(e1490)V*	10	47.37	14	50
**Males**				
him-5(e1490)V	20	N.A	31	N.A
*sin-3(tm1276)*I; *him-5(e1490)V*	11	45	16	48.39

Supplementary Table S2. Comparative estimation of *sin-3* gene on various physiological parameters.

Phenotype	% change observed in *sin-3;him5* (mutant genotype) hermaphrodite w.r.t isogenic control#	% change observed in dsRNAi against *sin-3* and empty vector L4440 on wild-type *
Life Span	47	47
Brood Size	37.5	~35.8
Body Length (YA stage)	15	~12
AVID	30	~27

The data presented is mean of the three independent experiments. Please refer materials and methods for details. * denotes that the dsRNAi treatment was carried out for three generations, following which the experiments were conducted.

Supplementary Table S3. Loss of *sin*-3 results in defective reproduction in the worms.

Genotype	No. of eggs	No. of animals hatched	% hatching	Number of male progeny	% males
**Self mating**					
him-5(e1490)V	253	252.2	99.68	116	46
*sin-3(tm1276)*I;*him-5(e1490)V*	115	81.2	70.60	30	37.3
**Mating**					
him-5(e1490)V	271	268	98.89	116	43.59
*sin-3(tm1276)*I;*him-5(e1490)V*	101	74.6	73.86	31	37.5

Supplementary Table S4. *sin-3* deletion results in increase body length.

Time after L4 (h)	*sin-3; him-5* (Hermaphrodite)		*him-5 (e1490)* (Hermaphrodite)	
	Mean (µm)	SEM	Mean (µm)	SEM
0	812.33	4.63	698.66	2.02
24	1015.66	3.38	878.33	5.45
48	1378.00	3.78	1031.00	3.05
72	1621.00	5.13	1233.66	2.90
96	1858.66	12.99	1259.33	1.76

Table represents the mean ± SEM body length after designated hours of attaining stage L4. The body length has been measured in µm.
